# Understanding Viral Transmission Behavior via Protein Intrinsic Disorder Prediction: Coronaviruses

**DOI:** 10.1155/2012/738590

**Published:** 2012-10-14

**Authors:** Gerard Kian-Meng Goh, A. Keith Dunker, Vladimir N. Uversky

**Affiliations:** ^1^Center for Computational Biology and Bioinformatics, Indiana University School of Medicine, Indianapolis, IN 46202, USA; ^2^Department of Biochemistry, Yong Loo Lin School of Medicine, National University of Singapore, Singapore 119228; ^3^Department of Molecular Medicine, University of South Florida, Tampa, FL 33612, USA; ^4^Institute for Biological Instrumentation, Russian Academy of Sciences, 142290 Pushchino, Russia

## Abstract

Besides being a common threat to farm animals and poultry, coronavirus (CoV) was responsible for the human severe acute respiratory syndrome (SARS) epidemic in 2002–4. However, many aspects of CoV behavior, including modes of its transmission, are yet to be fully understood. We show that the amount and the peculiarities of distribution of the protein intrinsic disorder in the viral shell can be used for the efficient analysis of the behavior and transmission modes of CoV. The proposed model allows categorization of the various CoVs by the peculiarities of disorder distribution in their membrane (M) and nucleocapsid (N). This categorization enables quick identification of viruses with similar behaviors in transmission, regardless of genetic proximity. Based on this analysis, an empirical model for predicting the viral transmission behavior is developed. This model is able to explain some behavioral aspects of important coronaviruses that previously were not fully understood. The new predictor can be a useful tool for better epidemiological, clinical, and structural understanding of behavior of both newly emerging viruses and viruses that have been known for a long time. A potentially new vaccine strategy could involve searches for viral strains that are characterized by the evolutionary misfit between the peculiarities of the disorder distribution in their shells and their behavior.

## 1. Introduction

### 1.1. Protein Intrinsic Disorder and Viral Behavior

Previously, we provided evidence that the behavior of viruses can be predicted from the analysis of their predicted intrinsic disorder in their protein shells, more specifically, by looking at the peculiarities of disorder distribution in their matrix and capsid proteins [1–3]. For example, the predicted disorder in the matrix of retroviruses was shown to vary with the mode of the viral transmission. The HIV and EIAV viruses, that are related but have distinctly different modes of transmission, were used to illustrate this point since. HIV is largely transmitted sexually, whereas EIAV is transmitted by a blood-sucking horsefly. It has been observed that the abundance of predicted intrinsic disorder (PID) in the HIV and EIAV matrix proteins was very different, with the HIV proteins being highly disordered, especially HIV-1. An explanation for this has to do with the need for a more rigid encasement in viruses that are not sexually transmitted, so as to protect the virion from harsher environmental factors [1].

### 1.2. Goals

Further development of a model that could predict how a virus will behave in terms of transmission would be extremely useful for both clinical and fundamental research. Such a model will also provide a tool to assist the implementation of public health policies for handling old and newly emerging pathogenic viruses. This paper extends the line of research on protein intrinsic disorder in viral proteins to coronaviruses (CoV), which have caught the attention of the scientific community because of the sudden appearance of the lethal virus causing severe acute respiratory syndrome, the SARS-CoV [4, 5]. Clinical, structural, and epidemiological data are available for SARS-CoV and its animal cousins, which remain to be a serious threat to farming communities [4, 6–10].

One goal of this research is to use of the concept of protein intrinsic disorder to shed light on behaviors of coronaviruses by creating a predictive model, which could provide insight into the differences between the transmission behavior of animal and human coronaviruses and also classify the various animal coronaviruses by their spread behavior. In this way, greater understanding of the viral evolution based on its hosts and its environment can be achieved along with the better understanding of the structural mechanisms involved in such adaptive evolution.

### 1.3. Coronaviruses

For a long time, coronaviruses have been known to cause respiratory diseases and gastroenteritis [4]. Greater attention was given to this family of viruses when the SARS-CoV moved into human hosts beginning in 2002 and inflicted 1091 deaths [11, 12]. During the outbreak of SARS-CoV, questions regarding its modes of transmission were raised [7, 13]. Greater understanding of the peculiarities of the transmission mode(s) would have enabled better decisions to control the spread of this ominous virus. However, as of today, complete understanding of the molecular mechanisms determining the transmission behavior of the virus remains elusive [6]. For example, there is no satisfactory explanation for the observation that many of those infected with SARS-CoV did not have any contact with the infected individuals [14].

While SARS-CoV had been observed to spread among human most easily by respiratory means, the SARS-CoV was also observed to be not as infectious as Influenza (http://www.who.int/csr/sars/sarsfaq/en/) [5]. It is likely that SARS-CoV, like most animal coronaviruses, spreads most efficiently by contact or by oral-fecal routes among animals.

Several human coronaviruses (HCoV) such as 229E, OC43, HKU1, and NL63 have been known for some time [15, 16], with HCoV-229E being the most studied HCoV. Comparison between the spread mechanisms of HCoV and animal coronaviruses such as SARS-CoV has been made. Using HCoV-229E as a representative virus, it has been shown that HCoV tends to spread more efficiently by a respiratory route, whereas the animal CoVs tend to spread most efficiently by direct contact [15]. Furthermore, gastroenteritis is a common disease provoked by coronaviruses, and oral-fecal route is also common mode of transmission, especially among animal hosts. Therefore, in contrast to human, contact and oral-fecal routes are generally the most efficient mode of CoV spread in animals [15, 17, 18].

### 1.4. Structural Proteins

In order to introduce the two structural proteins investigated in this paper, a quick overview of virus structure needs to be put forth. Proteins near or at the surface of the coronavirus include the spike protein (S, 150 kD), the hemagglutinin-esterase protein (HE, 65 kD), membrane/matrix glycoprotein (M, 25 kDa), and the small envelope glycoprotein (E, 9–12 kD). A protein that is closer to the RNA is the nucleocapsid protein (N, 60 kD). They are all likely to play roles in protecting the virions [1, 2, 19, 20]. Since the M glycoproteins are the most abundant structural proteins in the coronavirus, they are likely to play a greater role in protecting the virion from damage.

The M-protein is a triple-spanning transmembrane protein and has a short aminoterminal ectodomain. Other than its protective function, M-protein also plays a role in the capsid self-assembly and serves as a major determinant of the virion morphogenesis via selecting the S protein for incorporation into virions during viral assembly. Another protein of interest to this paper is the N-protein [5, 12]. The N-protein is an RNA binding protein. While many of its functions remain unknown, it is likely that the N-protein is involved in packaging and protecting the viral RNA and also plays a role in viral replication participating in transcription [7, 8].

### 1.5. Grouping of Coronaviruses

Although human coronaviruses, with the exception of SARS-CoV, are generally mild, their animal cousins (e.g., the Avian, Porcine, and Bovine coronaviruses) are often devastating to the farming industry. In fact, an outbreak could be costly in terms of the livestock loss [10, 21]. Coronaviruses are generally classified into three groups based on their genetic and antigenic makeup [12, 16]. A summary of this classification is shown in Table 1. The SAR-CoV did not fit into any of the current three groups but did have some resemblance to Group 2. For this reason, SARS-CoV fell into a new category, group 2b.

Previously, we showed that the similarity in protein intrinsic disorder prediction does not necessarily reflect the genetic proximity of proteins studied, but rather could be related to the evolution, the modes of transmission, and the environment that the virus lives in [1, 2]. Therefore, the categorization of coronaviruses based on the intrinsic disorder propensities of their proteins might provide important clues for useful hypotheses, especially when intrinsic disorder of the similar proteins is considered across coronaviruses, serotypes, or subtypes.

### 1.6. Protein Intrinsic Disorder

The main tools used here are the predictors of protein intrinsic disorder. Intrinsically disordered proteins have also been described by various other names such as “intrinsically unstructured” [22] and “natively unfolded” [23, 24]. The generality of the intrinsic disorder concept emerged from the occasional observations of exceptions to the structure-to-function paradigm according to which unique protein structures are necessary for specific protein functions [25]. Comparison of ordered and disordered proteins revealed that there is a noticeable difference between the amino acid sequences of ordered and intrinsically disordered proteins and that disordered proteins/regions share at least some common sequence features over many proteins [24, 26–28]. In fact, the disordered proteins/regions were shown to be significantly depleted in bulky hydrophobic (Ile, Leu, and Val) and aromatic amino acid residues (Trp, Tyr, and Phe), which would normally form the hydrophobic core of a folded globular protein, and also possess low content of Cys and Asn residues. The depletion of disordered protein in Cys is also crucial as this amino acid residue is known to have a significant contribution to the protein conformation stability via the disulfide bond formation or being involved in coordination of different prosthetic groups. These depleted residues, Trp, Tyr, Phe, Ile, Leu, Val, Cys, and Asn, were proposed to be called order-promoting amino acids. On the other hand, ID proteins were shown to be substantially enriched in Ala, polar, disorder-promoting amino acids: Arg, Gly, Gln, Ser, Glu, and Lys and also in the hydrophobic, but structure-breaking Pro [27–31]. Development of such predictors provided a direct support for the hypothesis that intrinsic disorder is encoded in protein amino acid sequences. These features made intrinsically disordered proteins/regions recognizable and were used to develop specific predictors of intrinsic disorder. Currently, design of algorithms for finding regions lacking ordered structure is a very active area of research, and more than 50 predictors of disorder have been developed [32]. The predictor used in this paper is PONDR VLXT (Predictors of Naturally Disordered Regions), which is a set of neural network predictors of disordered regions on the basis of local amino acid composition, flexibility, hydropathy, and other factors [33–36]. These predictors classify each residue within a sequence as either ordered or disordered. Since PONDR VLXT is sensitive to local sequence peculiarities, it is frequently used for identifying functionally important sites within the disordered regions.

## 2. Results

### 2.1. General Trends of Viral Predicted Disorder

**Figure 1:**
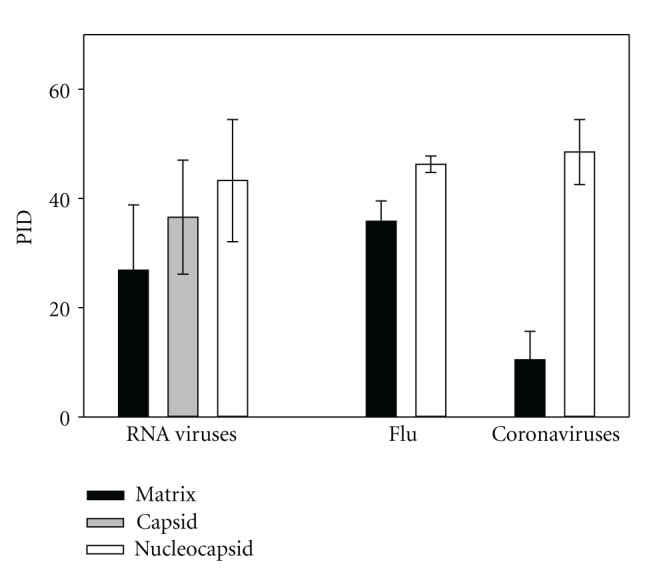
A comparison of the mean PID of membrane (matrix glycoprotein, M) and nucleocapsid (N) proteins of coronavisruses with those of the influenza A virus and RNA viruses in general. Similarities and dissimilarities can be observed.

Figure 1 represents a graphical comparison of the percent of intrinsic disorder (PID) of the M- and N-proteins of coronaviruses with that of the influenza A virus and the matrix and nucleocapsid proteins of RNA viruses in general. The mean PIDs of matrix and nucleocapsid proteins of the influenza A virus and coronaviruses provide an interesting contrast since both viruses have similarities and dissimilarities. For example, the amounts of disorder in the N-proteins of both viruses are rather similar, whereas the M-proteins of coronaviruses are predicted to be more ordered. Since the major function of M-proteins is to protect the virion, it is tempting to hypothesize that these differences in the overall disorder of M-proteins can be related to the need to protect viruses from different environments, and therefore can reflect differences in the viral transmission mode. We know, for example, that both viruses are often spread via droplet transmission, but animal coronaviruses are often more associated with oral-fecal transmission routes. The differences in the PIDs may reflect this trend.

**Figure 2:**
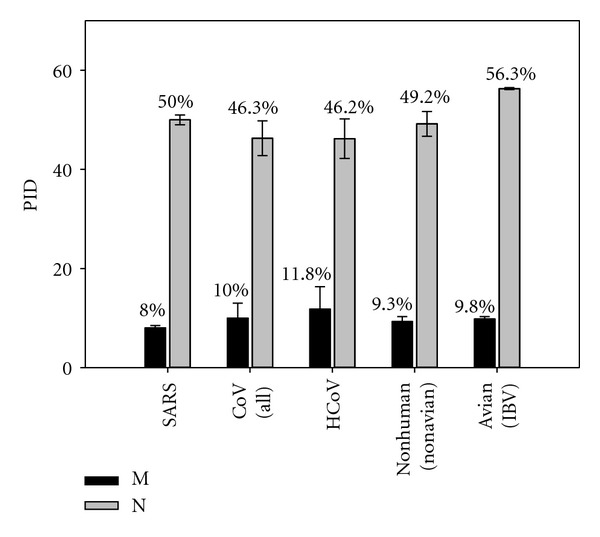
Comparison of the mean PID of membrane (matrix glycoprotein, M) and nucleocapsid (N) proteins of human and nonhuman coronaviruses. Mean PIDs in the N- and M-proteins of avian coronavirus are shown for comparison.

A more detailed comparison between the PID in M- and N-proteins of the HCoVs and those of animal coronaviruses in terms of predicted disorder is shown in Figure 2. Avian and SARS coronaviruses are also included for comparison. Figure 2 shows that the N-protein of the avian coronavirus, IBV, is characterized by the highest PID level. The high PID variance (standard deviation) of the HCoV M- and N-proteins should be also noted. Both features likely hint to a higher respiratory spread component.

### 2.2. Disorder of Nucleocapsid and Matrix Proteins of Coronaviruses Varies with the Type of the Human and Animal Hosts

**Figure 3:**
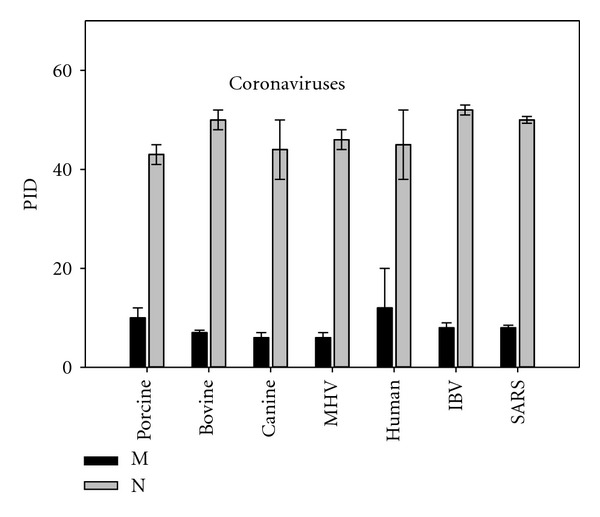
The mean PIDs of the M- and N-proteins of human coronaviruses (HCoV) and the various animal coronaviruses including avian coronavirus.

While Figure 2 allows us to compare human coronaviruses (HCoV) with nonhuman ones in general, Figure 3 breaks down the data of animal coronaviruses further by animal hosts. We are able to see differences in PID of both matrix and nucleocapsid proteins of viruses by the various animal host species.

**Figure 4:**
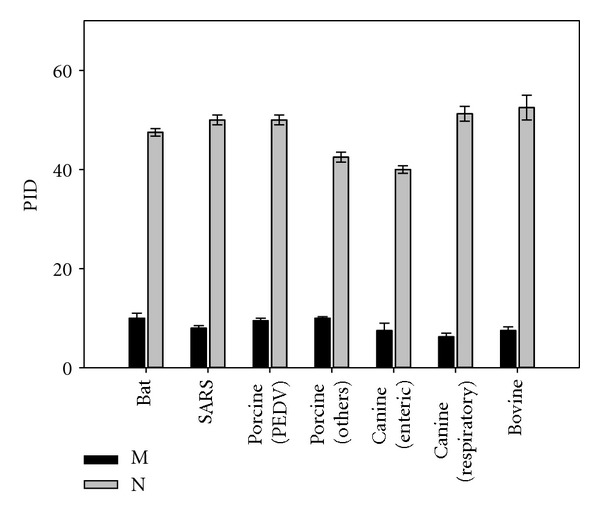
The mean PIDs of the M- and N-proteins of the various animal coronaviruses. Strains of porcine and canine coronaviruses are shown. Differences are seen between the strains of porcine and canine coronaviruses.

Figure 4 provides an overview of the mean PIDs in the M- and N-proteins of various animal coronaviruses by species of the hosts and by the type of virus. The differences in PIDs between host types should be noted. For instance, in comparison with its porcine counterparts, bovine coronaviruses tend to have higher PIDs in their N-proteins. The important note here is that the PIDs of the N-proteins for all these animal coronaviruses are below 50%, unlike human or avian coronaviruses.

Table 2 provides numerical PID values for the N- and M-proteins of various porcine strains shown in Figure 4. A close examination of these data revealed that the particular strains may be quite different from each other by the amount of intrinsic disorder they possess in their N- and M-proteins. An illustrative example is the remarkable differences in the PIDs of the N-proteins from TEGV and PEDV. Here, the CV777 strain of the PEDV has lower disorder levels in its matrix protein than that of the Br1/87 strain (8% versus 13%). Using SARS-CoV as a reference point, we could clearly see that each strain of the virus is characterized by a unique PID signature. For example, TGEV is characterized by the relatively low PID score for its N-protein (~43%), whereas its M-protein has a somewhat higher PID (~14%) especially when compared to certain strains of PEDV (~8%).

### 2.3. Human Coronaviruses (HCoV)

**Figure 5:**
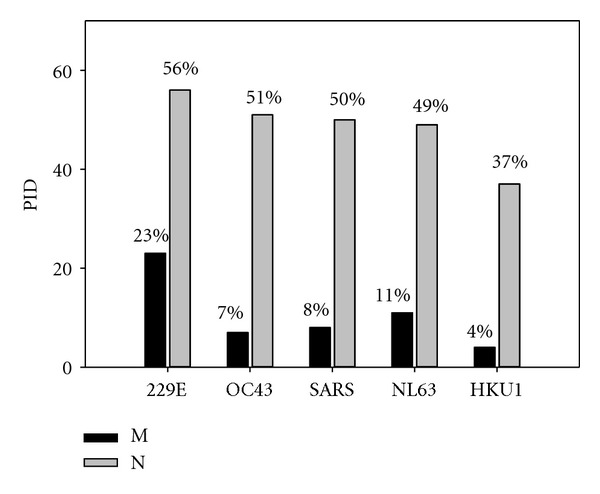
The mean PIDs in different strains of human coronavirus (HCoV) as compared to the SARS-CoV.

A breakdown of the HCoV PIDs by strains is shown in Figure 5 which shows the unusually high and low PIDs for the M- and N-proteins of the HCoVs 229E and HKU1. The noticeable differences in the PID values of HCoV 229E and SARS-CoV should also be noted. Some HCoV strains were studied clinically and the peculiarities of their transmission modes are listed in Table 1. The HCoV-229E has been studied more extensively than other human coronaviruses and has been shown to spread by respiratory modes more easily. This is also believed to be generally the case for all the HCoVs. The results shown in Figure 5 illustrate that, in general, the N- and M-proteins of human coronaviruses tend to be more disordered than those of the animal coronaviruses (cf. Figures 1–3).

Figure 5 also shows that both matrix and nucleocapsid proteins of the HCoV-229E are exceptionally disordered. Figure 5 also illustrates an important point that not all HCoV strains have the same characteristics as 299E and there is great variability in the PIDs of the matrix and nucleocapsid proteins of HCoVs. For example, the predicted disorder in HKU1 for some reason resembles the mean PIDs we would normally find in the animal coronaviruses.

### 2.4. Categorization of Coronaviruses by Shell Disorder and Transmission Behavior

The patterns of predicted disorder seen in Figures 1–5 allowed us to regroup the various coronaviruses by their PID levels.  Table 3 summarizes the new grouping of coronaviruses. It could be seen that although the new grouping of CoVs shown in Table 3 is based on the disorder analysis of the viral shells, and in particular their N-proteins, the grouping seems to reflect how the viruses spread. In other words, a correlation between common transmission behavior and the disorder characteristics of the viruses in each group can be observed. We see, for example, that based on their PID analysis, the canine respiratory coronavirus falls in Category B, which contains viruses with moderate levels of respiratory and fecal-oral components. Its less disordered enteric counterpart falls into Category C, which has greater fecal-oral component and less respiratory component. This pattern is also seen for the porcine coronaviruses TGEV and PEDV.

Therefore, data shown in Table 3 can be used for the prediction of the levels of respiratory and oral-fecal transmission modes for each virus in a group based on the PIDs of their M- and N-proteins. For example, we can conclude that viruses in Group A will have greater respiratory component than those in Group B or C. MANOVA analysis reveals statistically significant influence of the PIDs of both N and M proteins on the transmission behaviour of coronaviruses (*P* < 0.05 for each variable, *F* = 16.3, *P* < 0.05). Furthermore, because of the statistical significances of the model and its variables, the three groups should be easily identifiable via the analysis of the PID levels of the corresponding M and N proteins. However, any correlation between PIDs of the two proteins is not statistically significant (MANOVA, *P* = 0.15). An interesting note is that not all the viruses in the Category C have low PID levels of their M-proteins, even if their N-proteins are characterized by low predicted disorder.

**Figure 6:**
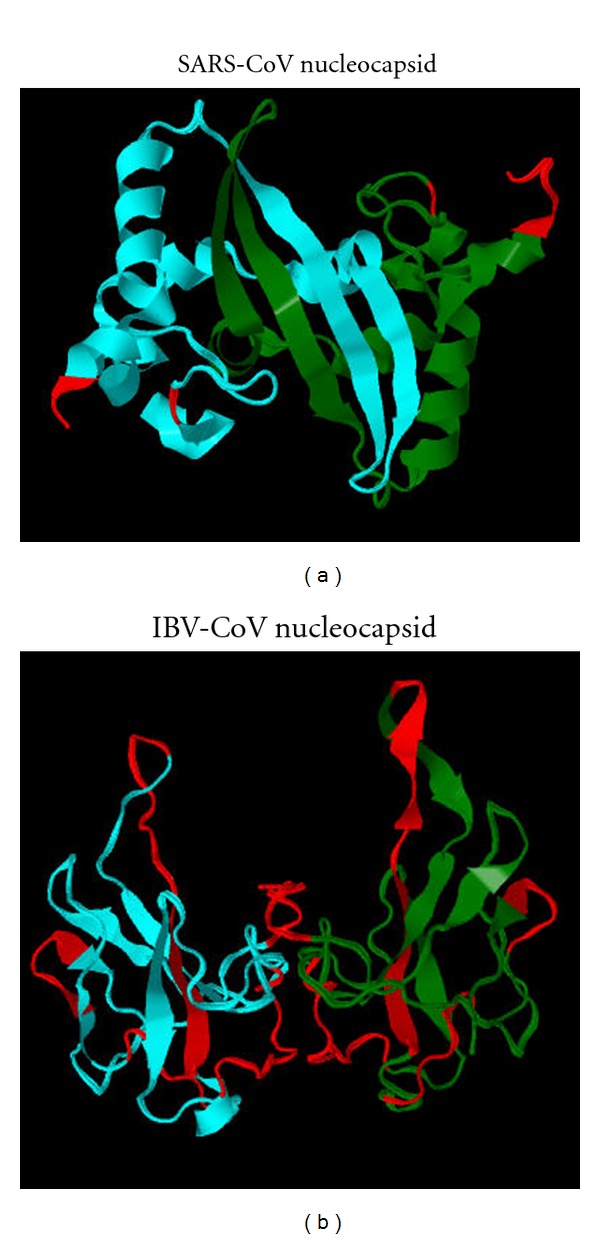
3D representation with predicted disorder annotation of the parts of the nucleocapsid proteins of IBV and SARS-CoV [7, 8]. (a) SARS-CoV (PDB ID: 2gib); (b) IBV (PDB ID: 2c86) Avian infectious bronchitis virus (IBV). The red color denotes residues predicted to be disordered by PONDR VLXT, while cyan and green represent regions that are predicted to be ordered with the two colors used to distinguish between separate subunits. The mean PID of the IBV N-protein is 56%, whereas the corresponding PID of SARS-CoV N-protein is only 50% (see Table 3). The N-protein of HCoV-299E is more similar to the IBV N-protein in this respect.

While the figures and tables above show the differences in mean PID levels between different CoV proteins, Figure 6 visualizes the differences in the predicted disorder levels by comparing the 3D structures of parts of the N-proteins of SARS-CoV and IBV. Large disordered regions (shown in red) can be seen in the IBV nucleocapsid, whereas the SARS-CoV nucleocapsid possesses much smaller amount of disordered regions. A rough disorder analysis revealed that the PID of the SARS-CoV nucleocapsid is 50%, whereas in the case of IBV nucleocapsid, the mean PID is of 56%. Figure 6 suggests that this difference may be reflected on their structural differences.

## 3. Discussion

### 3.1. Predicted Disorder Varies with the Evolution and Transmission Mode of the Virus

#### 3.1.1. Protective Functions of the Viral Shells: Stepwise Disorder towards the Core

Data shown in Figure 1 suggest that the protective functions of the viral surface proteins can be seen from the results of protein disorder predictions. In fact, there is a correlation between the PID levels and the localization of surface proteins, with proteins located closer to the virion surface being generally more rigid than other surface proteins (e.g., in coronaviruses, nucleocapsid proteins possess noticeably higher PID than the matrix proteins). Generally, the stepwise decrease in the PIDs is seen for all viral nucleocapsid, capsid, and matrix proteins analyzed so far.

#### 3.1.2. Disorder in the Nucleocapsid Associates with Infectivity Pathways Especially for Viruses Transmitted via the Respiratory Mode

Figure 1 shows that there are similarities and dissimilarities between the PID levels in the influenza and coronavirus shell proteins. Disorder peculiarities can be potentially mapped to the transmission behavior and the nature of the viruses. It can be seen that in comparison with other viruses, the coronaviruses possess a tendency to be more ordered at the level of the matrix proteins, but their nucleocapsid proteins are generally more disordered than nucleocapsid proteins RNA viruses in general. The fact that both influenza viruses and coronaviruses have high predicted disorder in their nucleocapsid proteins suggests that higher nucleocapsid disorder may be necessary for viruses with ability to spread via respiratory means. Further support for this hypothesis will be presented below as we inspect and categorize the various coronaviruses. Past analysis showed that many viruses that spread by respiratory modes are characterized by disordered shell proteins [3].

#### 3.1.3. Higher Shell Rigidity, Higher Levels of the Oral-Fecal Transmission Mode

Viral shell proteins constitute the protective proteinaceous layer which is needed for virus to survive during its transmission between the hosts. Figure 1 shows that the mean PID levels of M-proteins in coronaviruses are strikingly lower than those of influenza A. On the other hand, coronaviruses are often transmitted via the oral-fecal and fecal-contact modes [10, 13, 15, 18], whereas the major transmission mechanism of the influenza viruses is the respiratory mode. Based on these and similar observations we hypothesize that there is a correlation between the PID levels in the viral shell proteins and the virus transmission modes. While higher levels of disorder in shell proteins are likely to be associated with greater probability of respiratory transmission, lower PID levels in the nucleocapsid and matrix proteins are conversely often associated with increased levels of fecal-oral transmission mode. These correlations can be due to the need of a given virus to adjust to the changes in the environmental conditions associated with the process of transmission between the hosts.

Obviously, the environmental conditions associated with the respiratory transmission are less harsh than those seeing in the fecal-oral transmission mode. However, the viruses that are spread via the respiratory mode might experience greater environmental pressure during transmission and they have to be able to adjust to a greater range of environmental changes to survive and to be successfully transmitted. This ability of the respiratory transmitted viruses to adjust and survive in a wide range of conditions can be due to the higher levels of intrinsic disorder in their shell proteins. In other words, shell proteins of these viruses are assembled into a flexible shield, the pliability of which helps viruses to rapidly adjust to the environmental changes.  Table 3 supports this hypothesis by showing that for several viruses analyzed, there is a noticeable correlation between the level of disorder in the shell proteins and the transmission mode.

### 3.2. Patterns of Protein Intrinsic Disorder Might Determine the Peculiarities of the Animal Infection by Coronaviruses

Disorder patterns are linked to the behavior of the viruses and their hosts. Marked differences in disorder of shell proteins were predicted among human, avian, and other animal coronaviruses. There are also noticeable differences in the disorder propensities among the coronaviruses infecting different animal (Figures 2–6, Table 2). For example, the PID levels in the matrix and nucleocapsid proteins of the porcine and canine coronaviruses are generally lower than those of the bovine counterparts (Figure 3). A plausible explanation lies in the nature of the hosts of the various viruses and in the mode of the virus transmission. Furthermore, since the habits and diets of the various animals can also contribute to the tendency of them to be exposed to fecal material, that are crucial factors determining the peculiarities of the virus transmission. Both the porcine and canine coronaviruses have greater fecal-oral components, whereas the bovine coronaviruses are more efficiently transmitted via the respiratory routes. All these factors are apparently more definitive determinants of the intrinsic disorder levels in the viral shell proteins than the genetic proximities and the categorization of the viruses seen in Table 1.

#### 3.2.1. Differences in Intrinsic Disorder Seen in Different Coronaviruses Infecting the Same Host Are Potentially Linked to the Types of the Infected Organs

Some variations in the levels of predicted intrinsic disorder might depend not only on the transmission mode but also on the nature of organ affected by the virus during infection. Figure 4 shows that the canine respiratory coronavirus (that affects the respiratory tract) has higher PID levels than the canine enteric coronavirus (preferentially affecting intestines). Similar patterns can also be seen among the porcine coronaviruses (see Table 3).

### 3.3. Porcine Coronaviruses: A Path towards Understanding of the Correlation between the Protein Disorder and Viral Behavior

#### 3.3.1. Some Enigmatic Characteristics of Porcine Coronaviruses

The transmissible gastroenteritis coronavirus (TGEV) and porcine epidemic diarrhea virus (PEDV) have similar modes of transmission. Although they are preferentially passed on via the oral mode, both viruses can be transmitted through the lungs too [17, 18], with the PEDV being generally less efficiently transmitted. A puzzling aspect of the PEDV is that this virus has the ability to reemerge to infect hosts months after the breeding areas have been cleaned [17]. This suggests that PEDV is perhaps more persistent in the environment outside the host organism. As it will be seen from the next paragraphs, our analysis suggested that intrinsic disorder can produce useful information for better understanding of the viral behavior.

#### 3.3.2. The Use of the Intrinsic Disorder Predictions to Address Some Viral Behavior Puzzles

Tables 2-3 show that the level of predicted disorder in the TGEV N-protein is noticeably lower than that of the PEDV analogue, and that at least one PEDV strain has a noticeably lower PID in its M-protein (Table 2). Based on the fact that the M-protein of PEDV possesses the greater rigidity we hypothesize that some strains of PEDV are likely to be more persistent outside the physiological environments when compared to TGEV. This hypothesis is also consistent with the observed PEDV ability to survive in the harsh nonphysiological environment (outside the host), to sustain the disinfection of the pig pens and infect new hosts many months after the first infection [17].

#### 3.3.3. Protein Shell Disorder Can Explain Mechanisms of Infection and Persistence

Our analysis also suggests that the observed intrinsic disorder propensities of the PEDV shell proteins may be related to the way of how the virus spreads between hosts. Because of the higher levels of intrinsic disorder predicted in the N-protein by PONDR VLXT, it is expected that there is a relatively large “respiratory” component in the PEDV transmission (see Table 3). On the other hand, TGEV spreads more easily since contact and oral-fecal modes are the more advantageous forms of transmission among swine [17, 18].

#### 3.3.4. SARS-CoV and PEDV Possess Similar Transmission Behaviors and Comparable Levels of Predicted Disorder

The comparison of the PID levels in the M- and N-proteins suggests that various coronaviruses can be grouped as shown in Table 3. An important result of this analysis is the conclusion that in terms of the intrinsic disorder in M- and N-proteins of various coronaviruses, the closest neighbor of the SARS-CoV is PEDV. The structural disorder similarity suggests that the behaviors of these two viruses might also be similar, insights that were not previously noticed.

#### 3.3.5. Evolutionary Misfits Are Potential Targets for a New Vaccine

It should be noted, however, that there are some strains of the animal coronaviruses that could be used as counterexamples of the categorization shown in Table 3. One of such case is porcine respiratory coronavirus (PRCV), which is a mild variant of the transmissible gastroenteritis coronavirus (TGEV) that has been observed to transmit predominantly by respiratory means and can scarcely be detected in feces or small intestines [4, 10]. The PRCV transmission behavior was explained by the specific changes in the viral spike (S protein) which defined the ability of this protein to interact with specialized cells in the respiratory tract, and not with the receptors found in cells from the gastrointestinal region [10]. Since PRCV and TGEV have virtually identical levels of predicted disorder in their M- and N-proteins, both of them should be assigned to the Category C, that is, to the class of coronaviruses that are spread mostly via the fecal-oral and contact modes. Therefore, PRCV that relies on the respiratory transmission mode may be inept by evolution. This potential evolutionary misfit may also explain why the symptoms resulting from the PRCV infection are generally mild and why PRCV is a vaccine strain. The observation may also suggest a new vaccine search strategy based on the recognition of viruses or strains that are evolutionary misfits.

### 3.4. Disorder Comparison among HCoV, SARS-CoV, and Other Animal Coronaviruses Provides Clues to Viral Behavior

#### 3.4.1. Large Variations Are Seen for Disorder in Human Coronaviruses: Complexity of Human Immune Systems

Data shown in Figures 1–5 and Table 3 can be used for the comparison of the human matrix and nucleocapsid proteins with the corresponding proteins from the nonhuman coronaviruses. A striking feature is the presence of large PID variability for both the M and N of human coronaviruses especially when compared to those of nonhuman animal coronaviruses. This difference is likely due to the greater complexity of the human immune system [37]. Furthermore, many of the HCoVs are characterized by the relatively high levels of predicted disorder in their N and M proteins (see Table 3 and Figure 5). Since our analysis revealed that higher PID levels in one or both of the capsid proteins typically correspond to the coronaviruses with greater respiratory transmission component, the high disorder levels seen for both M- and N-proteins may indicate that many HCoVs are more easily spread by respiratory means, an important hypothesis which is in agreement with the clinically and experimentally observed transmission behavior of HCoVs [15].

#### 3.4.2. Similarities and Differences between the Avian IBV-CoV and Human HCoV-229E

Figures 2–6 and Table 3 show that nucleocapsid of the avian coronavirus (IBV) is characterized by the high predicted disorder levels. The only virus that possesses the comparable level of disorder is the human coronavirus 229E (Figures 3–5). However, HCoV-229E and IBV-CoV have some differences too. In HCoV-229E, both M- and N-proteins are disordered, whereas in IBV-CoV, the nucleocapsid is more disordered but the PID of the M is only slightly above average. This implies that although IBV-CoV and some HCoV are likely to be highly infectious mostly via the respiratory transmission, the IBV virus could also be present in the gastrointestinal region, and larger quantity of this virus can be found in stools.

#### 3.4.3. Greater Disorder in Avian Coronavirus Nucleocapsid: Greater Respiratory Component

Previous studies revealed that predicted intrinsic disorder in the matrix and surface proteins of other avian viruses is often associated with the peculiarities of their evasion of immune system [1, 2, 38]. Here, the ability of the viruses to evade the immune systems may allow them to be spread between the different host species. Another reason for the high disorder in the avian influenza nucleocapsid is related to its predominant transmission via the respiratory modes. Comparison of the PID values in N- and M-proteins of these two viruses showed that despite the comparable disorder levels at the nucleocapsid, the M-protein of IBV-CiV is predicted to be relatively more ordered than the corresponding protein of the influenza virus (Figure 2). This is an indication of the greater likelihood of the presence of the virus in stools, despite its predominant respiratory transmission. In agreement with this hypothesis, IBV-CoV has been reported to be present in feces of infected birds, and respiratory mode is believed to be the main transmission means [21, 39]. This may point to the greater possibility of fecal-respiratory transmission in IBV. Similar interpolations can also be made using the analysis of the various porcine coronaviruses in terms of their shell disorder and their respective transmission behavior.

#### 3.4.4. Characteristic Differences between HCoV and Other Animal Coronaviruses

Based on the experimental and clinical studies on the HCoV-229E and SARS-CoV, it has been concluded that HCoV-229E is easily transmissible by respiratory modes, whereas SARS-CoV is more easily transmitted via contact [15], and these observations were used to generalize the transmission differences between human and other coronaviruses [15]. Our data on protein intrinsic disorder support this conclusion and also provide some additional points for consideration.

#### 3.4.5. Higher Respiratory Component in Many but Not All HCoVs

The fact that HCoVs are generally characterized by high PID levels of their M-proteins suggests that many human coronaviruses may have a higher “respiratory” component in their transmission mode. However, as we have seen above, the variability of predicted disorder in HCoV is very high, with HCoV-229E possessing the highest PID rates among HCoVs and with HCoV-HKU1 being characterized by lowest levels of predicted disorder in its N- and M-proteins. This suggests that it is difficult to expect that all the HCoVs would have the same transmission characteristics as HCoV-229E. In fact, some HCoVs resemble nonhuman coronaviruses. For example, HCoV-HKU1 is quite different from other HCoVs, even though several of them are found in the same group (see Table 1 and Figure 5).  Table 3 further emphasizes the presence of a noticeable variability in the PID levels of HCoV surface proteins and also shows that different HCoVs can have noticeable variability in their transmission modes.

#### 3.4.6. HCoV-HKU1 and MHV: An Enigmatic Relationship

It has been generally believed that HCoV-HKU1 is spread by respiratory means like the other HCoVs. However, more recent studies revealed that HCoV-HKU1 may be quite different from the other HCoVs. For example, there is a noticeable difference between the genetic makeups of HCoV-HKU1 and HCoV-OC43, and, genetically, HCoV-HKU1 seems to be close to the MHV. Furthermore, a case was described where HCoV-HKU1 was detected in a patient suffering from hepatitis [40]. Given this observation and the close genetic proximity of HCoV-HKU1 and MHV, it is suspected that HCoV-HKU1 may play a role in the pathogenesis of hepatitis. In agreement with these observations and hypotheses, Table 3 shows that there is a close similarity in the PID levels evaluated for the surface proteins of HCoV-HKU1 and MHV. This finding is rather surprising, even if the genetic similarity of these two CoVs were to be taken into account, since data shown in Tables 1–3 clearly illustrate that genetic proximity does not necessarily translate into the disorder pattern similarity. This also suggests that HCoV-HKU1 and MHV may also possess similar transmission behavior. This hypothesis is supported by the recent clinical studies which indicated the greater presence of HCoV-HKU1 in stools of patients and the greater association of gastrointestinal illness with HCoV-HKU1 infections when compared to cases of other HCoV infections [41]. Viruses, such as MHV, that infect the liver may need harder shell encasements to protect the virion during their exposure to bile, which is known to inflict damage on certain viruses [42].

#### 3.4.7. Moderate Disorder in SARS-CoV and PEDV-CoV When Compared to HCoV-229E

Based on the results of the disorder analysis, we can hypothesize that there is a great likelihood that HCoV-229E is most easily spread via the respiratory mode. Based on the PID data shown in Table 3 one can expect that SARS-CoV should have a larger fecal-oral component than HCoV-229E and therefore should likely be spread by contact, oral-fecal, and respiratory means. In fact, more moderate PID levels in SARS-CoV and many animal CoVs imply that these viruses are likely to have reasonably high levels of both oral-fecal and respiratory components, which allow them to persist in the environment for a longer time and yet being able to infect via droplets or aerosols. These hypotheses are consistent with the previously described direct observations of the mostly respiratory transmission mode of HCoV-229E [15]. On the other hand, although many animal CoVs were shown to have a larger fecal-oral component, they also can be spread by touch, since a larger fecal-oral component will allow them to persist in the environment for a longer time, or via the fecal-respiratory mode.

### 3.5. SARS-CoV: Understanding the Transmission Behavior of Coronaviruses via Predicted Disorder

#### 3.5.1. The SARS-CoV Puzzles

One of the difficulties that epidemiologists experienced during the SARS outbreak of 2003-4 was the way the virus spreads. From the way the virus passed between the hosts, it was known that the virus has some “respiratory” components already at the beginning of the outbreak. When it was then shown to be a coronavirus, a special attention was paid to possible oral-fecal routes, given the nature of coronaviruses. In fact, there were instances where the spread involved fecal routes, such as in the case of the outbreak at Amoy Gardens in Hong Kong, which involved a spread via the sewerage system [11, 43]. Despite of what was known from the outbreak at Amoy Gardens, the exact modes of SARS-CoV spread remain unclear as of yet [6]. For example, there were many cases where patients were infected without any known contact with an infected person [14]. Therefore, the precise spread mechanisms remain somewhat a mystery, and there are no appropriate animal models for the SARS-CoV transmission modes.

#### 3.5.2. SARS-CoV and PEDV: Fecal-Respiratory Routes

The disorder analysis of SARS-CoV and other coronaviruses suggests that SARS-CoV falls in Category B (see Table 3), which includes viruses that are likely to have intermediate levels of both respiratory and fecal-oral components. These viruses are expected to have less respiratory component than HCoV-229E and IBV from the Category A (Table 3 and Figures 3, 5, and 6), but greater contribution of the respiratory mode than viruses of the Category C, such as TGEV and MHV. By the same token, the SARS-CoV has a higher oral-fecal component than the former but lower oral-fecal component than the latter. This suggests a greater likelihood of being spread via the fecal-respiratory mode, which affirms what has been long suspected clinically. The role of fecal-respiratory transmission, as suggested by the disorder analysis mentioned above, also explains many of features observed for PEDV, and not observed for its counterpart, TGEV. It also explains similarly in the spreading behaviors of SARS-CoV and PEDV, given that both have the closest shell disorder seen in our sample.

#### 3.5.3. SARS-CoV: Signs of Persistence Seen in the Disorder Analysis Data

Another feature that was a puzzle during the SARS epidemic and remains unclear now is the question on the SARS-CoV persistence outside the physiological environment. Although the ability of SARS-CoV to survive on the surfaces and feces for extended periods is known [6, 43], the level of persistence and the corresponding evolutionary mechanisms have not been fully understood. The results shown in Tables 2-3 suggest a way to address the problem. In the case of PEDV and SARS-CoV, the PID levels of both N- and M-proteins are quite moderate. In PEDV and TGEV, the differences in the transmission behavior and the levels of shell disorder suggest that special attention should be paid to the PID peculiarities of the M-protein. A sign of persistence of SARS-CoV and some specific strains of PEDV (see Tables 2-3) can be found in the relatively low PID levels of their M-proteins (~8%), especially when compared to viruses such as TGEV (~14%) and HCoV-229E (~23%).

#### 3.5.4. Infections without Contact with Infected Hosts

Another enigma is the ability of SARS-CoV to infect hosts without any apparent contact with an infected host [6, 44]. A comprehensive understanding of the mechanisms involved in this ability remains elusive. The differences between PEDV and TGEV in terms of shell disorder and observed spread characteristics offer some clues to this conundrum, since the ability of PEDV to infect pigs remains even after attempted cleaning of pens and isolation of infected pigs [17]. Both shell proteins of both PEDV and SARS-CoV are predicted to be moderately ordered. Therefore, the same reasoning used to predict the environmental persistence and the greater fecal-respiratory transmission component in PEDV can be extended to explain the behavior of SARS-CoV.

#### 3.5.5. Spread Inefficiency of SARS-CoV and PEDV

Another similarity between the SARS-CoV and PEDV is their relative transmission inefficiency. The SARS-CoV did not exhibit higher spread efficiency especially when compared to influenza A, whereas PEDV has been observed to spread less quickly than TGEV. It would then be tempting to conclude that these inefficiencies are for the same reasons. A closer analysis of the corresponding intrinsic disorder data reveals that the reasons for the spread inefficiency are actually very different. Comparisons of the PIDs of N-proteins of SARS-CoV (Tables 2-3), IBV (Figure 6), and HCoV-229E (Figure 5, Table 2) revealed that the intrinsic disorder in the SARS-CoV N-protein is relatively moderate. Conversely, when the N-protein of SARS-CoV is compared to TGEV (Tables 2-3) and many other animal CoVs, the opposite is held true (Figure 2): the PID level in the SARS-CoV N-protein is higher than the corresponding levels in many animal coronaviruses, especially TGEV.

## 4. Conclusions

### 4.1. Predicting Viral Behaviors Using a Model Based on the Analysis of the Rigidity of the Shell Proteins

A model to relate disorder at the M- and N-proteins to the behavior of coronaviruses can be built. This model is based on the observation that viruses that are exposed to a less harsh environment, such as those that are transmitted via oral-fecal route, require a more rigid encasement so as to protect their virions from damage. One way to measure the rigidity of the encasement is by looking at the level of predicted intrinsic disorder in the capsid and matrix proteins. From the known behavior and shell disorder patterns of influenza A virus and various porcine coronaviruses, it can be deduced that higher disorder at M-protein can be linked to greater persistence, while higher (or lower) levels of predicted disorder in the nucleocapsid can be related to the levels of respiratory (or fecal-oral) transmission components, respectively. While the disorder levels in these two proteins tend to follow each other, this is not necessarily the case for all coronaviruses.

### 4.2. Observed and Predicted Evidence of the Respiratory Spread in HCoV-229E and IBV-CoV

Previous studies have already provided evidence that sexually transmitted viruses often have highly disordered M- and N-proteins since such viruses do not need a rigid protective shell but, instead, flexible layers of proteins that could help these viruses evade the host's immune system. In this paper, a number of coronaviruses were sampled, and further support of the link was found. For instance, many CoVs that are known to spread predominantly by the respiratory mode are characterized by higher disorder levels in their shell proteins. This is especially the case for the HCoV-229E, for which our analysis revealed that both the M- and N-proteins are highly disordered. The data also suggest that many other human coronaviruses may rely on the respiratory route as the main mode of transmission since HCoVs generally have large PID levels in both matrix and nucleocapsid. An especially large respiratory component has been observed for HCoV-229E when compared with animal coronaviruses including the SARS-CoV [15]. Such high level of disorder was not generally detected in the animal CoVs, with the exception of the avian IBV. It is likely that IBV possesses higher ability to spread via fecal-respiratory route given the more ordered M-protein and more disordered nucleocapsid protein.

### 4.3. Behavioral Similarities of PEDV and SARS-CoV

The results of the disorder analysis of the shell proteins of the porcine coronaviruses not only gave more evidence to link predicted disorder to viral behavior but also showed how the model can be used to predict the CoV behavior. In fact, there is a close similarity between the PID levels in the matrix and nucleocapsid proteins of SARS-CoV and PEDV. Furthermore, the transmission behaviors of these two viruses are strikingly similar (both PEDV and SARS-CoV spread primarily by contact), despite their relatively distant genetic relationship (Table 1). On the other hand, based on the lower nucleocapsid PID found in TGEV, we could expect to see a larger oral-fecal component in the transmission of this virus. This suggests that since TGEV has much larger oral-fecal and contact components in transmission, it can spread much more efficiently than PEDV, the hypothesis that can be deduced from the presented PID analysis. This suggests that the levels of predicted disorder provide a definite way to evaluate the fitness of the virus.

### 4.4. PEDV and SARS-CoV: Higher Levels of the Aerosol Spread

The model also suggests a mechanism by which PEDV could reemerge to infect herds even after cleaning of the premises [17]. Our analysis suggests that this ability to reemerge arises from the existence of both oral-fecal and respiratory components. Therefore, the aerosol transmission arising from the viral particles left behind may have a crucial role in this reemerging. In its turn, the ability to have two transmission components can be determined by the peculiarities of the predicted intrinsic disorder distribution in the viral shell proteins. PID similarity between PEDV and SARS-CoV suggests that these two viruses might have similar transmission mechanisms and suggests that SARS-CoV might have propensity to reemerge, which can explain how people could be infected even though they had not been known to be in contact with the SARS-CoV-infected persons [14]. This model also suggests that reemerging behavior cannot be expected for TEGV. Overall, the disorder-based analysis constitutes the categorization algorithm shown in Table 3 that can be used to identify the transmission modes of various coronaviruses.

### 4.5. A New Tool for Understanding Behavior of Existing and Newly Emerging Viruses

Results of our analysis show that disorder predictors can be used to predict transmission behavior of newly emerging strains of viruses. In the cases of epidemic outbreaks, such a technique would be a useful tool for predicting the potential transmission behavior of a new virus. Furthermore, this analysis gives new clues for better understanding the spread behavior of viruses that have been known for years.

## 5. Materials and Methods

Searches for the appropriate samples were made using the Entrez website.  Table 4 represents some of the proteins analyzed in this study. Proteins with known X-ray crystal structures were chosen to ensure consistency. Further details of implementation can be found in our previous papers [1, 2, 38]. The data extracted from PDB were placed into the database that enables further development of the JAVA codes to create 3D representation in Jmol [45]. The relational database was modified to allow import and storage of the data from UNIPROT (http://www.uniprot.org/) [46]. A graphical user interface program was implemented using JAVA to allow easy entry of protein details and accession codes.

The open-source statistical package, R (http://www.r-project.org/), was used to analyze data. Statistical methods used include MANOVA (Multivariate Analysis of Variance).

## Figures and Tables

**Table 1:** Categorization of various animal coronaviruses of interests by genetic and antigenic proximity [4, 5, 16]. The viruses included are porcine transmissible gastroenteritis virus (TGEV), porcine epidemic diarrhea virus (PEDV), and human coronaviruses (HCoV).

Groups	Coronaviruses	Diseases caused
	Human-HCov 229E and NL63	Pneumonia colds
1	Porcine-transmissible gastroenteritis coronavirus (TGEV) and porcine epidemic diarrhea virus (PEDV)	Gastroenteritis, pneumonia
	Bat	Respiratory enteritis

2	Human-HCoV OC43E and HKU1	Respiratory
	Canine coronavirus (CCoV)	Gastroenteritis
	Bovine coronavirus (BCoV)	Gastroenteritis, pneumonia
	Murine hepatitis virus (MHV)	Encephalitis, hepatitis

2b	SARS CoV	Pneumonia, gastroenteritis

3	Avian infectious bronchitis virus (IBV)	Respiratory, kidney

**Table 2:** The PID values in the M- and N-proteins of various porcine coronaviruses and SARS-CoV. The SARS-CoV values are shown here for the comparison.

Coronavirus	Strain	PID (%) in M-proteins	PID (%) in N-proteins	UniProt Accession (M)	UniProt Accession (N)
TGEV	FS772/70	14	43	P09175	P05991
PEDV	CV777	8	51	P59771	Q07499
PEDV	Br1/87	13	NA	P59770	NA
SARS	NA	8	50	P59596	P59595

**Table 3:** A new categorization of coronaviruses by predicted disorder.

Disorder group	Coronavirus	PID (M)	PID (N)	Remarks for the group
A	IBV (Avian) HCoV-229E	9.8 23	56 56	The most disordered group. Respiratory transmission. IBV matrix protein is more ordered than HCoV-229E.

B	SARS-CoV PEDV Bovine Canine (resp.) Bat HCoV-OC43 HCoV-NL63	8 8 7.4 6.5 11.5 7 11	50 51 53 51 47 51 49	Moderately disordered group. Some fecal-oral and contact transmissions component. Some respiratory transmission. TGEV has a low nucleocapsid PID but unusually high matrix PID. However, because of the unusually low nucleocapsid disorder, TGEV is placed in group C.

C	TGEV MHV Canine (Ent.) HCov-HKU1	14 8 8 4	43 47 40 37	The least disordered group. Higher levels of fecal-oral and contact transmission. Lesser levels of the respiratory transmission component.

**Table 4:** Some of the coronavirus proteins analyzed in this study.

Disorder group	Coronavirus	PID (M)	PID (N)	UniProt ID (M)	UniProt ID (N)	PDB ID (M)	PDB ID (N)
A	IBV(Avian) HCOV-229E	9.8 23	56 56	P11222 P15422	P32923 P15130		2ge8, 2gec

B	SARS-CoV PEDV Bovine Canine (Resp) Bat OC43 NL63	8 8 7.4 6.5 11.5 7 11	50 51 53 51 47 51 49	P59596 P59771 P69704 A3E2F6 A3EXD6 Q4VID2 Q6Q1R9	P59595 Q07499 Q8V432 A3E2F7 Q3LZX4 P33469 Q6Q1R8	3i6g	1ssk, 2jw8

C	TGEV MHV Canine (Ent.) HKU1	14 8 8 4	43 47 40 37	P09175 Q9JEB4 B8RIR2 Q14EA7	P04134 P03416 Q04700 Q0ZME3		3hd4

## References

[1] Goh GKM, Uversky VN, Dunker AK (2008). A comparative analysis of viral matrix proteins using disorder predictors. *Virology Journal*.

[2] Goh GKM, Dunker AK, Uversky VN (2008). Protein intrinsic disorder toolbox for comparative analysis of viral proteins. *BMC Genomics*.

[3] Xue B, Williams RW, Oldfield CJ, Goh GKM, Dunker AK, Uversky VN (2010). Viral disorder or disordered viruses: Do viral proteins possess unique features?. *Protein and Peptide Letters*.

[4] Holmes KV (2003). SARS coronavirus: a new challenge for prevention and therapy. *Journal of Clinical Investigation*.

[5] Stadler K, Masignani V, Eickmann M (2003). SARS—beginning to understand a new virus. *Nature Reviews Microbiology*.

[6] Yu ITS, Sung JJY (2004). The epidemiology of the outbreak of severe acute respiratory syndrome (SARS) in Hong Kong—What we do know and what we don’t. *Epidemiology and Infection*.

[7] Yu IM, Oldham ML, Zhang J, Chen J (2006). Crystal structure of the severe acute respiratory syndrome (SARS) coronavirus nucleocapsid protein dimerization domain reveals evolutionary linkage between Corona- and Arteriviridae. *Journal of Biological Chemistry*.

[8] Jayaram H, Fan H, Bowman BR (2006). X-ray structures of the N- and C-terminal domains of a coronavirus nucleocapsid protein: implications for nucleocapsid formation. *Journal of Virology*.

[9] Peiris JSM, Chu CM, Cheng VCC (2003). Clinical progression and viral load in a community outbreak of coronavirus-associated SARS pneumonia: a prospective study. *The Lancet*.

[10] Saif LJ, Sestak K, Straw BE, Taylor DJ (2006). Transgastroenteritis and porcine respiratory coronaviruses. *Diseases of Swine*.

[11] Abraham T (2004). *Twenty-First Century Plague: The Story of SARS*.

[12] Groneberg DA, Hilgenfeld R, Zabel P (2005). Molecular mechanisms of severe acute respiratory syndrome (SARS). *Respiratory Research*.

[13] Christian MD, Poutanen SM, Loutfy MR, Muller MP, Low DE (2004). Severe acute respiratory syndrome. *Clinical Infectious Diseases*.

[14] Wu J, Xu F, Zhou W (2004). Risk factors for SARS among persons without Known Contact with SARS patients, Beijing, China. *Emerging Infectious Diseases*.

[15] Atmar R, McMillan JA, Feigin RD, DeAngelis C, Jones MD (2006). Coronaviruses. *OSki's Pediatrics: Principle and Practice*.

[16] Acheson N (2007). *Fundamental of Molecular Virology*.

[17] Pensaert MB, Yeo S, Straw BE, Taylor DJ (2006). Porcine epidemic diarrhea. *Diseases of Swine*.

[18] Sestak K, Saif LJ, Gonzales AM, Monila A, Yoon K, Zimmerman JJ (2002). Porcine coronaviruses. *Trends in Emerging Viral Infections of Swine*.

[19] Kao CC, Ni P, Hema M, Huang X, Dragnea B (2011). The coat protein leads the way: an update on basic and applied studies with the Brome mosaic virus coat protein. *Molecular Plant Pathology*.

[20] Ehrlich LS, Liu T, Scarlata S, Chu B, Carter CA (2001). HIV-1 capsid protein forms spherical (immature-like) and tubular (mature-like) particles in vitro: structure switching by pH-induced conformational changes. *Biophysical Journal*.

[21] Cook JK, Pattison M, McMullin J, Bradbury J (2008). Coronaviruses. *Poultry Diseases*.

[22] Wright PE, Dyson HJ (1999). Intrinsically unstructured proteins: re-assessing the protein structure-function paradigm. *Journal of Molecular Biology*.

[23] Weinreb PH, Zhen W, Poon AW, Conway KA, Lansbury PT (1996). NACP, a protein implicated in Alzheimer’s disease and learning, is natively unfolded. *Biochemistry*.

[24] Uversky VN, Gillespie JR, Fink AL (2000). Why are “natively unfolded” proteins unstructured under physiologic conditions?. *Proteins: Structure, Function and Genetics*.

[25] Uversky VN, Dunker AK (2010). Understanding protein non-folding. *Biochimica et Biophysica Acta*.

[26] Dunker AK, Lawson JD, Brown CJ (2001). Intrinsically disordered protein. *Journal of Molecular Graphics and Modelling*.

[27] Vacic V, Uversky VN, Dunker AK, Lonardi S (2007). Composition Profiler: a tool for discovery and visualization of amino acid composition differences. *BMC Bioinformatics*.

[28] Radivojac P, Iakoucheva LM, Oldfield CJ, Obradovic Z, Uversky VN, Dunker AK (2007). Intrinsic disorder and functional proteomics. *Biophysical Journal*.

[29] Williams RM, Obradovi Z, Mathura V (2001). The protein non-folding problem: amino acid determinants of intrinsic order and disorder. *Pacific Symposium on Biocomputing*.

[30] Romero P, Obradovic Z, Li X, Garner EC, Brown CJ, Dunker AK (2001). Sequence complexity of disordered protein. *Proteins*.

[31] Dunker AK, Lawson JD, Brown CJ (2001). Intrinsically disordered protein. *Journal of Molecular Graphics and Modelling*.

[32] He B, Wang K, Liu Y, Xue B, Uversky VN, Dunker AK (2009). Predicting intrinsic disorder in proteins: an overview. *Cell Research*.

[33] Vucetic S, Brown CJ, Dunker AK, Obradovic Z (2003). Flavors of protein disorder. *Proteins*.

[34] Romero P, Obradovic Z, Kissinger CR (1998). Thousands of proteins likely to have long disordered regions. *Pacific Symposium on Biocomputing*.

[35] Garner E, Romero P, Dunker AK, Brown C, Obradovic Z (1999). Predicting binding regions within disordered proteins. *Genome Informatics*.

[36] Obradovic Z, Peng K, Vucetic S, Radivojac P, Brown CJ, Dunker AK (2003). Predicting intrinsic disorder from amino acid sequence. *Proteins*.

[37] Perelson AS, Bragg JG, Wiegel FW (2006). The complexity of the immune system: scaling laws. *Complex Systems Science in Biomedicine*.

[38] Goh GKM, Dunker AK, Uversky VN (2009). Protein intrinsic disorder and influenza virulence: the 1918 H1N1 and H5N1 viruses. *Virology Journal*.

[39] Ignjatović J, Sapats S (2000). Avian infectious bronchitis virus. *OIE Revue Scientifique et Technique*.

[40] Esper F, Weibel C, Ferguson D, Landry ML, Kahn JS (2006). Coronavirus HKU1 infection in the United States. *Emerging Infectious Diseases*.

[41] Esper F, Ou Z, Huang YT (2010). Human coronaviruses are uncommon in patients with gastrointestinal illness. *Journal of Clinical Virology*.

[42] Smith W (2005). The action of bile salts on viruses. *The Journal of Pathology and Bacteriology*.

[43] Yu ITS, Li Y, Wong TW (2004). Evidence of airborne transmission of the severe acute respiratory syndrome virus. *New England Journal of Medicine*.

[44] Wu J, Xu F, Zhou W (2004). Risk factors for SARS among persons without known contact with SARS patients, Beijing, China. *Emerging Infectious Diseases*.

[45] Herráez A (2006). Biomolecules in the computer: jmol to the rescue. *Biochemistry and Molecular Biology Education*.

[46] Apweiler R, Bairoch A, Wu CH (2004). UniProt: the universal protein knowledgebase. *Nucleic Acids Research*.

